# Animal Shelters’ Response to the COVID-19 Pandemic: A Pilot Survey of 14 Shelters in the Northeastern United States

**DOI:** 10.3390/ani11092669

**Published:** 2021-09-10

**Authors:** Lauren Powell, Caitlin Houlihan, Martha Stone, Ilana Gitlin, Xuke Ji, Chelsea L. Reinhard, Brittany Watson

**Affiliations:** School of Veterinary Medicine, University of Pennsylvania, Philadelphia, PA 19104, USA; chouli@vet.upenn.edu (C.H.); mestone@vet.upenn.edu (M.S.); igitlin@vet.upenn.edu (I.G.); xukeji@vet.upenn.edu (X.J.); creinh@vet.upenn.edu (C.L.R.); brittawa@vet.upenn.edu (B.W.)

**Keywords:** animal shelter, COVID-19, pandemic, adoption, foster care, euthanasia, dog, cat

## Abstract

**Simple Summary:**

During the COVID-19 pandemic, there were widespread reports of increased public interest in adopting and providing foster care to pets in animal shelters. However, there is a need for peer-reviewed scientific evidence to support these trends. The goals of this preliminary study were to investigate possible differences in the number of animals entering and exiting animal shelters in the Northeastern United States and describe changes that shelters made to their usual operations in response to COVID-19. Fourteen animal shelters completed an anonymous, online survey between 2 November and 31 December 2020. Fewer dogs and cats were admitted to animal shelters and adopted between March–June 2020 compared with the same months of 2019. We found that the proportion of animals who were adopted or euthanized did not differ between the years, although there were considerable differences between the shelters. While many shelters endeavored to recruit new foster caregivers during the pandemic, the overall proportion of animals who spent time in foster care was no greater in 2020 compared with 2019. Our study provides pilot data about how the COVID-19 pandemic affected animal shelter operations and illustrates the range of different experiences of animal shelters in the Northeastern United States.

**Abstract:**

Anecdotal reports indicate that many animal shelters experienced increased adoption and foster care rates during the COVID-19 pandemic, yet peer-reviewed evidence is lacking. In this pilot survey of 14 animal shelters in the Northeastern United States, we aimed to investigate the impact of the COVID-19 pandemic on animal intakes, foster care and five outcome types and describe operational changes reported by shelters in response to COVID-19. Paired sample *t*-tests and Wilcoxon signed-rank tests were used to compare intake, adoption, euthanasia and foster care rates and numbers between March–June 2019 and 2020. The number of dogs and cats that entered shelters was significantly lower during the COVID-19 pandemic compared with the same months of 2019 (*t* = 3.41, *p* = 0.01, *t* = 2.69, *p* = 0.02). Although the overall rate of adoption and euthanasia did not differ, the numbers adopted and euthanized decreased significantly for both dogs and cats, reflecting the significantly decreased intake. We also found significant variability between shelters. During the pandemic, several shelters sought to expand their foster care networks through operational changes (*n* = 6) and statements made to the public (*n* = 7). However, the proportion of dogs and cats housed in foster care did not differ between March–June 2019 and 2020 in our sample. Our findings offer preliminary insights regarding the impact of a worldwide pandemic on the functioning of animal shelters.

## 1. Introduction

The World Health Organization (WHO) declared the COVID-19 outbreak a global pandemic on 11 March 2020 [1]. SARS-CoV-2, the causative agent of COVID-19, is primarily spread between humans. Physical distancing and isolation were therefore employed as key strategies to reduce viral transmission worldwide, particularly in the early stages of the pandemic before the widespread use of face masks and the availability of vaccines [2,3]. In the United States, each state had the authority to implement their own physical distancing policies, so the COVID-19 response varied substantially between states. Most states in the Northeastern United States implemented stay-at-home orders in mid to late March 2020 [4] and the statewide closures in the region were some of the longest lasting in the country [5].

Veterinary activities were widely recognized as essential services and many animal shelters continued to function during the pandemic [6,7]. The U.S. Centers for Disease Control and Prevention (CDC) in collaboration with industry leaders, such as the American Veterinary Medical Association (AVMA), developed guidelines to help animal shelters continue operating during the pandemic and protect the health of shelter staff and the public [8]. Academic shelter medicine programs also released recommendations for safe operating practices. Strategies included reducing non-essential intake of animals (e.g., intake of healthy kittens), limiting or discontinuing non-essential services (e.g., routine spay/neuter), and utilizing appointments for most services (e.g., adoptions) [9]. Animal shelters were also advised to offer community support services to help pet owners retain their pets during the pandemic, such as in-home care or temporary housing in the shelter [8,9]. It is currently unclear how animal shelters applied these recommendations and what operational changes, if any, were employed.

The COVID-19 pandemic may also have directly affected the number of animals entering and exiting animal shelters. Although COVID-19 is spread almost exclusively through human-to-human transmission, cases of zoonotic transfer have been documented [10]. Preliminary studies showed that dogs and cats could be infected with SARS-CoV-2 both naturally and experimentally, although cats appeared to be more susceptible to symptomatic infection and viral shedding [11,12,13]. More recent evidence suggests human–pet transmission of SARS-CoV-2 may be more common than initially thought, with data indicating that 20–67% of companion cats and dogs became infected with COVID-19 following exposure [14,15,16,17]. The possibility of zoonotic transfer of COVID-19 lead to fears that animal shelters may see a spike in relinquishment rates, particularly in the early stages of the pandemic [18]. Financial strain, housing difficulties and the emergence of new behavioral problems during the pandemic also had the potential to increase relinquishment [19]. To date, these fears appear to have been unsubstantiated. In their study of an Israeli pet adoption website, Morgan et al. [20] found that the rate of relinquishment was stable throughout the lockdown period and in the following months as businesses began to reopen.

Interest in pet ownership also increased during the COVID-19 pandemic. Ho et al. [21] reported Google searches for pet adoption, dog adoption and cat adoption increased by up to 250% compared with 2019, with peak interest occurring between April and May 2020. Research has shown that the rates of dog adoption and foster care applications also increased drastically in Israel relative to the start of the pandemic [20]. Most dog owners in the study indicated that they were planning to acquire a dog regardless of COVID-19 but were motivated to adopt during the pandemic as they had extra time available. Some owners were also motivated to adopt a dog to reduce their stress and loneliness, or due to (seemingly misleading) reports of dog abandonment [20]. The news media has described similar increases in dog adoption rates and foster care applications within the U.S. [22,23]. A news release by the AVMA also showed higher rates of adoption and foster care relative to intake in 2020 compared with 2019 [24]. However, peer-reviewed literature is limited, and further research is needed to describe adoption trends and animal shelters’ experiences during COVID-19. This pilot study aimed to investigate the initial impact of the COVID-19 pandemic on animal intake and outcomes at shelters in the Northeastern United States and to describe the shelters’ operational changes in response to COVID-19.

## 2. Materials and Methods

### 2.1. Participants

Study participants were recruited between 2 November and 31 December 2020, through social media postings and email discussion lists for animal shelters and veterinarians. Emails were also sent directly to eligible animal shelters where an email address was publicly available. To be eligible to participate in this study, shelters had to have a physical brick-and-mortar facility, run an adoption program, and operate in the Northeastern area of the United States (Delaware, Pennsylvania, New Jersey, New York, Connecticut, Rhode Island, Massachusetts, New Hampshire, Vermont, Maine, or Maryland). One representative staff member was instructed to complete the survey on behalf of their shelter. The representative staff member was required to have a deep understanding of the vision and mission of the institution, decision-making power, and access to shelter data, such as an executive director or shelter manager. Participants were also encouraged to share the survey with other eligible animal shelters.

One hundred nineteen individuals started the survey, although a total of 105 respondents were excluded as they did not provide data regarding their intakes and outcomes (*n* = 93), their shelter was located outside the eligible states (*n* = 11), or they did not have an adoption program (*n* = 1). Fourteen shelters were included in the final sample. All participants were over the age of 18 and provided written informed consent to participate in this study. This study received exempt approval status from the Institutional Review Board of the University of Pennsylvania.

### 2.2. Survey

The survey was administered using Qualtrics (Qualtrics, Provo, UT, USA) and all responses were recorded anonymously. The survey comprised 29 questions and took approximately 30 min to complete. It included descriptive questions about the shelter’s location (state of operation, rural/urban/suburban), admission type, funding type and the respondent’s position. Shelters were then asked to provide data regarding the number of animals entering their facility (owner surrender, stray, transfer in), the animals’ outcomes (adopted, return to owner, return to field, transfer out, euthanized) and the average length of stay for dogs and cats housed in their facility or in foster homes between March–June 2019 and 2020 based on outcome date. We also included questions about the shelter’s programs and operations, including which programs were operating between March–June 2019 and 2020, the shelter’s motivations for implementing operational changes and the sources used to guide operational changes. Finally, there was an open-text question in which shelters could describe anything else relevant to their response during the COVID-19 pandemic.

### 2.3. Statistical Analysis

Descriptive statistics were calculated for intakes and outcomes. Histograms were used to assess the normality of the data. Adoption and euthanasia rates were calculated as the proportion of total intake adopted or euthanized. Foster care rates were calculated as the number of animals that spent time in foster care relative to the total shelter population. Paired sample *t*-tests were used to compare intake, housing (foster/exclusively in shelter) and adoption rates (relative to intake) between March–June 2019 and 2020. As the data for euthanasia rates (relative to intake), foster care rates (relative to total shelter population) and specific intake types were not normally distributed, Wilcoxon signed-rank tests were used to compare March–June 2019 and 2020. Frequency data were used to describe operational changes between 2019 and 2020. All analyses were conducted using SPSS (IBM SPSS Statistics for Windows, version 27, Armonk, NY, USA). Statistical significance was set at *p* < 0.05.

## 3. Results

Fourteen representative individuals completed the survey on behalf of their shelter, including eight executive director/CEOs, two shelter managers, one chief officer, one director of operations, one deputy director, and one receptionist. The descriptive characteristics of the shelters are shown in Table 1.

### 3.1. Intake and Outcomes

Intake and outcome data for March–June 2019 and 2020 are shown in Table 2. Dog intake was 45% lower in March–June 2020 with a mean intake of 137 dogs (range 20–290) compared with 248 dogs (range 46–583) during the same period in 2019 (*t*(11) = 3.41, *p* = 0.01), with significant decreases seen across all intake categories (*p* ≤ 0.03). Similarly, total cat intake decreased by 29% from a mean of 373 (range 48–900) in March–June 2019 to 264 (range 56–691) in March–June 2020 (*t*(13) = 2.69, *p* = 0.02). In particular, the number of cats who were relinquished by their owners was significantly lower in 2020 compared with 2019 (*Z* = −2.45, *p* = 0.01).

**Figure 1:**
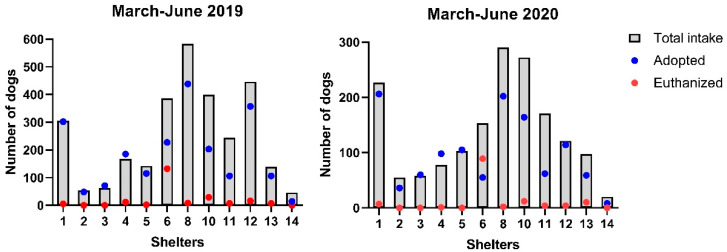
Dog intake, adoption, and euthanasia rates.

**Figure 2:**
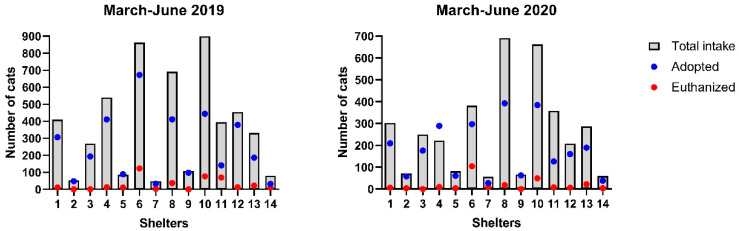
Cat intake, adoption, and euthanasia rates.

The number of dogs and cats that were adopted and euthanized decreased significantly from 2019 to 2020 (Table 2), although there were no significant differences in the proportion (rate) adopted or euthanized relative to intake (*p* ≥ 0.48, Figure 1 and Figure 2). For example, the mean number of dogs and cats adopted decreased by 46% and 28% in March–June 2020 compared with 2019, although the proportion of intake that was adopted did not change significantly. The mean dog adoption rate was 76% (SD 26%) in March–June 2019 and 74% (SD 29%) in 2020 and for cats, the mean adoption rate was 70% (SD 20%) in March–June 2019 and 72% (SD 22%) in March–June 2020. Similarly, we found a 39% decrease in the number of dogs and cats euthanized between 2019 and 2020, although the rates of euthanasia relative to intake were not significantly different. The mean euthanasia rate for dogs was 5% (SD 9%) in 2019 and 7% (SD 16%) in 2020, while the mean euthanasia rate for cats was 7% (SD 6%) in 2019 and 6% (SD 7%) in 2020.

However, there was considerable variability between shelters. Seven shelters reported canine adoption rates that were 7–39% lower in 2020 compared with 2019, while five shelters reported adoption rates that were 13–31% higher in 2020 (Supplementary Table S1). Five shelters reported little change in their feline adoption rates between 2019 and 2020, while five shelters reported decreases of 8% to 30% and four shelters reported increases in their feline adoption rates of 17–71% (Supplementary Table S2). Three shelters (shelters 4, 10 and 14) had increased rates of adoptions of both dogs and cats in 2020.

The number (*t*(8) = 0.83, *p* = 0.43, *t*(9) = −0.59, *p* = 0.57) and proportion (*Z* = −0.17, *p* = 0.87 and *Z* = 1.86, *p* = 0.06) of dogs and cats housed in foster care were comparable between 2019 and 2020. The mean percentage of dogs that were housed in foster care was 26% (SD 34%) in 2019 and 26% (SD 33%) in 2020, and for cats, the mean percentage housed in foster care was 31% (SD 34%) in 2019 and 35% (SD 34%). Again, there was notable variation between shelters (Supplementary Tables S1 and S2). Three shelters reported an increased percentage of dogs housed in foster care during the pandemic, two of which did not house any dogs in foster care in the same months of 2019. Five shelters increased the proportion of cats housed in foster care, one of which only started housing cats in foster care in 2020. Conversely, four shelters reported decreased rates of canine foster care in 2020 and one shelter reported decreased rates of feline foster care.

### 3.2. Changes in Operations, Protocols and/or Programs Due to COVID-19

All shelters with available data indicated that they implemented changes to their operations, protocols and/or programs in response to the COVID-19 pandemic. Shelters reported that they primarily implemented changes to decrease the number of animal care staff (*n* = 8/11 shelters, 73%), volunteers (*n* = 8/11 shelters, 73%), and pets in shelters (*n* = 7/11 shelters, 73%) and to increase the number of foster homes (*n* = 6/11 shelters, 55%). A summary of these changes is available in Table 3. Supplementary Tables S3 and S4 provide more detailed information regarding each shelter’s operational changes between 2019 and 2020. Shelters consulted the following sources to manage their pandemic response: CDC guidelines (*n* = 11/11, 100%), industry statements, e.g., AVMA (*n* = 10/11, 91%), state/local government recommendations (*n* = 9/11, 82%), non-profit group statements, e.g., American Society for the Prevention of Cruelty to Animals (ASPCA, *n* = 8/11, 73%), university statements (*n* = 5/11, 46%), and staff at peer shelters (*n* = 2/11, 18%). Only one shelter had a written disaster or emergency response plan in 2019 and two shelters had begun to prepare written disaster plans in 2020. All shelters with available data made statements to the public describing why their operational changes were necessary (*n* = 11), seven out of the 11 shelters (64%) made public statements to ask for community help to increase the number of foster homes, and three out of 11 shelters (27%) made statements about why certain populations of animals (e.g., healthy, adult community cats) should not be brought to the shelter.

### 3.3. Open-Text Responses

In the open-text response, one shelter indicated that they experienced increased requests for owner euthanasia during the pandemic as many owners were struggling to access full-service veterinarians. They also restricted intakes to emergencies only and encouraged owners to rehome privately to avoid animals entering the shelter.

## 4. Discussion

In this pilot study of animal shelters in the Northeastern United States, we investigated the impact of the COVID-19 pandemic on shelter operations. We found that total animal intake decreased significantly for cats and dogs from March–June 2019 to 2020, which mirrors data published by Shelter Animals Count (a collaborative database of U.S. animal shelters) [25], PetPoint (the most common animal management software in the United States) [26] and the AVMA [24] that also showed reduced animal intake. The majority of shelters in our sample made operational changes and public statements to decrease the number of animals housed in their facilities which could have contributed to the observed reduction in intake.

Contrary to preliminary concerns in the sheltering community, we saw decreased owner relinquishments of cats and dogs during the pandemic compared with the same months of 2019. This finding is congruent with data aggregated by PetPoint that showed a 20% decrease in cat relinquishments and a 24% in dog relinquishments in 2020 compared with 2019 [26]. Pet ownership has been associated with mental health benefits [27,28] and some studies conducted during COVID-19 showed that pet ownership buffered the negative mental health effects of the pandemic [29,30,31,32]. Therefore, in addition to the operational changes employed by shelters to decrease intake, it is also possible that pet owners were reluctant to relinquish their pets during the pandemic if they believed their pets provided a source of companionship or helped to reduce loneliness or stress. Lack of time is another common reason for relinquishment [33,34], and stay-at-home orders enacted during the pandemic may have provided some owners with an increased opportunity to spend time with their pets.

Fewer dogs entered shelters from transport/transfer sources during March–June 2020 compared with 2019. This result is expected as non-emergency pet relocation efforts may not have been considered essential services and therefore would have been subject to state and local travel restrictions during the pandemic [35]. We also found decreased intake of stray/unowned dogs during the pandemic, possibly because of the stay-at-home orders that may have reduced the chances of members of public finding stray animals. It is also possible that fewer animal control officers were available to pick-up stray animals during this period. Again, these findings reflect data published by PetPoint that showed significant decreases in transfers and stray animals in 2020 compared with 2019 [26].

We did not find a significant difference in the rate of adoption relative to intake between 2019 and 2020, despite reports of increased interest in pet adoption in the U.S. media [22,36] and previous research from Israel [20]. There were substantial methodological differences between our study and the previous study from Israel with may explain the incongruous findings. For example, we surveyed animal shelters, whereas Morgan, et al. [20] investigated adoptions through a national pet adoption website and may have captured animals that were rehomed privately or without entering a shelter. 

We also found that the COVID-19 pandemic affected individual shelter quite differently. Two shelters reported considerably higher rates of dog and cat adoptions during the pandemic, while seven shelters reported decreased rates of cat and dog adoptions, one shelter reported an increased proportion of dog adoptions but a decreased proportion of cat adoptions during the pandemic, and one shelter reported a decreased proportion of dog adoptions but an increased proportion of cat adoptions in 2020 compared with 2019. It is difficult to explain these differences as we did find consistent operational changes between shelters, with the one exception being an increased reliance on appointments. It is possible that the shelter’s characteristics and the characteristics of the community it served contributed to the variability between shelters. For example, the two shelters that reported increased rates of canine and feline adoptions were both suburban, limited-admission shelters. Shelters could also have implemented changes that were not captured by this survey. For example, some shelters have suggested that conducting meet-and-greets with prospective adopters in the carpark, rather than the shelter environment, was beneficial for long-term animals with poor kennel behavior as it potentially increased their chances of adoption [37].

However, the overall number of animals that were adopted in 2020 was significantly lower than 2019, likely due to decreased overall intake. The decreased number of adoptions may also be explained by operational changes, such as reduced opening hours, appointments for adoption procedures, a lack of adoption outreach events and an increased reliance on virtual adoption methods. It is possible that adoption interest only increased for subsections of the shelter population, such as puppies or small dogs. Demand for other animals that are typically harder to place, such as large dogs or those with known behavioral or medical challenges [38,39,40], may have remained unchanged. Prospective owners could have acquired pets through other sources, such as breeders. Most prospective owners consider adoption as the most ethical choice when acquiring a pet [41,42], although many also believe that breeding can be conducted ethically and that owners should have choices when considering where they want to obtain a pet [41]. Physical appearance and pedigree status have been consistently identified as key considerations for prospective owners when choosing an animal which may also have motivated some owners to seek animals from different sources [39,40,43].

We found that fewer animals were euthanized in 2020 than 2019, consistent with the decreased intake, although the shelters in this study had very low numbers of euthanasia in both 2019 and 2020. Euthanasia rates were comparable between March–June 2019 and 2020 and, for the most part, did not increase in a meaningful way during the pandemic. It is possible that during the pandemic, these shelters continued to take in a similar proportion of animals that were unsuitable for rehoming due to medical or behavioral concerns. The non-significant differences in euthanasia rates between the years could also reflect an anomaly of our sample, which, considering the time commitment necessary to complete the survey, was potentially skewed towards animal shelters with greater resources and staff time. It would be interesting to investigate animal outcomes relative to the COVID-19 pandemic timeline among a larger sample of animal shelters.

The media reported a surge in number of foster care volunteer applications, particularly in the early stages of the pandemic [44]. Here, we found that most shelters actively sought to recruit new foster caregivers, one shelter developed a waitlist of available foster caregivers, two shelters initiated finder-to-foster programs where members of the public who found unowned animals were encouraged to become foster caregivers and two shelters placed animals in foster care homes in 2020 that had not utilized foster care homes at all in the same months of 2019. It was therefore surprising to find the proportion of cats and dogs housed in foster care was not significantly different between 2019 and 2020. Given the preliminary nature of this study and the resultant small sample size, it is plausible that the absence of statistically significant differences reflects a Type II error. In other words, we may have failed to reject the null hypothesis although a true difference exists. Alternatively, it is possible that animal shelters’ efforts to recruit new foster caregivers did not result in a sustained expansion of their foster programs or an increased proportion of animals spending time in foster homes. Future studies including larger sample sizes are needed to understand the true impact of the COVID-19 pandemic on shelter foster care programs.

Interestingly, we found that more shelters placed an increased proportion of cats in foster care homes during the pandemic compared with dogs. For example, only one shelter housed a smaller proportion of cats in foster care in 2020 with a decrease of approximately 20% from 2019, whereas four shelters reported decreases in the proportion of dogs housed in foster care of 44–100%. Additional studies are needed to understand why there were different foster care rates between species. Although, it is possible that many foster caregivers volunteered for the first-time during the pandemic and first-time caregivers may have felt more confident caring for cats initially or that foster cats would be easier to integrate into their households.

Some shelters made changes to their community and pet retention programs during the pandemic. Pet food pantries were the most implemented program, occurring in more than 40% of shelters in this sample. Human food insecurity increased drastically during the pandemic [45,46] and food insecurity is thought to be correlated between humans and pets [47]. Prior to the COVID-19 pandemic, pet food banks were the most common community program offered by animal shelters [48]. Several shelters stopped providing subsidized veterinary care to owners during the pandemic, presumably to reduce non-essential services and abide by physical distancing guidelines. Access to veterinary care is an ongoing issue, particularly for underserved communities [49], and many pet owners found that access to veterinary care was more difficult during the pandemic [19]. It is not clear how the cessation of low-cost veterinary care impacted underserved pet owners, although future research is warranted. Underserved pet owners have historically been neglected by the veterinary field and often have a lack of trust in the profession, so it is possible that closure of these services could have long-lasting effects [50].

The animal shelters in this study consulted a range of sources to guide their pandemic response which is encouraging. All shelters referred to CDC guidelines, and a vast majority also followed recommendations from industry leaders, such as the AVMA. However, only one shelter had a written emergency plans to consult and two shelters had begun to prepare written disaster plans in March–June 2020, which highlights a gap in emergency preparedness and the need for animal sheltering organizations to plan their disaster response and develop all hazards emergency operations plans.

This study was a student-led, pilot study that provides some of the first peer-reviewed data regarding the impact of the COVID-19 pandemic on animal shelters during the first three months. However, there are several study limitations. The number of shelters who completed the survey was small and the generalizability of the findings is limited. We focused on the experience of small to medium shelters located in the Northeastern United States to avoid introducing variability from differing state responses. Further research is needed to capture the impact of the COVID-19 pandemic on shelters nationwide. The survey was also relatively time-intensive, and some questions had significant amounts of missing data. The length of the questionnaire and the depth of the data requested likely reduced the number of responses. Animal shelters typically have limited resources and staff are often time poor which may explain the number of unanswered questions. Our findings may also be affected by non-response bias, meaning shelters that responded to our survey may have systematically differed from shelters that did not respond. Finally, we cannot ascertain how the changes to shelter operations identified in this study may impact companion animals. For example, it is possible that the observed decrease in owner relinquishments at animal shelters may have coincided with increased requests for euthanasia at private veterinarians if owners believed they were unable to rehome or relinquish their pets. The decreased intake of stray/unowned animals during COVID-19 could also have led to higher populations of free-roaming animals.

## 5. Conclusions

Animal shelters in the Northeastern United States implemented a variety of operational changes to decrease the number of animals and people in their facilities and comply with public health directives early in the COVID-19 pandemic. The shelters in this pilot study reported fewer dogs and cats entering their facilities between March–June 2020 compared with the same period in 2019. The number of animals that were adopted and euthanized also decreased, in line with the decreased intake, although rates of adoption and euthanasia were not consistently different. Several shelters made operational changes in a bid to expand their foster care programs during the pandemic. However, there were no significant differences in the number of cats or dogs who spent time in foster care between 2019 and 2020. Our findings provide preliminary insights regarding the impact of a worldwide pandemic on the functioning of animal shelters.

## Figures and Tables

**Table 1:** Descriptive characteristics of shelters (*n* = 14).

Shelter	Date of Lockdown	State	Location	Admission Type ^a^	Funding ^b^	Annual Operating Expenses ($)	Dog Intake 2019	Cat Intake 2019	Foster Care Program ^c^	COVID-19-Positive Homes	
										Intake	Quarantine ^d^
1	16 March 2020	New Jersey	Suburban	Limited	-	2,600,000	932	1161	✓	X	-
2	20 March 2020	Pennsylvania	Rural	Open	501c3	132,000	159	143	✓	X	-
3	14 March 2020	Connecticut	-	-	501c3	868,557	250	800	✓	X	-
4	23 March 2020	Pennsylvania	Suburban	Limited	501c3, private	5,785,458	674	1884	✓	X	-
5	16 March 2020	New York	Suburban	Limited	501c3, government contract, private	880,000	382	434	✓	✓	✓
6	20 March 2020	Massachusetts	Urban	Open	501c3, private	3,500,000	1217	3296	✓	✓	✓
7	08 March 2020	New York	-	-	501c3	82,995	-	97	X	X	-
8	21 March 2020	New Jersey	-	Open	-	5,500,000	1497	2365	✓	✓	X
9	24 March 2020	Massachusetts	Rural	Open	501c3, private	106,340	-	387	✓	X	-
10	15 March 2020	Pennsylvania	Urban, surburban, rural	Open	501c3, government contract	2,000,000	1302	2797	✓	✓	✓
11	23 March 2020	Pennsylvania	-	Open	-	1,034,283	1087	1380	✓	X	-
12	31 March 2020	Maine	Suburban, rural	Open	501c3, government contract, private	3,085,295	1367	1656	N/A	X	-
13	20 March 2020	New York	Urban, rural	Open	501c3, private, municipally funded	1,292,034	998	382	N/A	X	-
14	N/A	New York	Suburban	Limited	501c3, private, government contract	408,000	151	217	N/A	X	-

^a^ Open admission shelters have largely unrestricted intake policies for all animals. Limited-admission shelters accept animals based on their individual criteria and mission, and are not obliged to take stray animals. ^b^ 501c3 indicates that the shelter has been approved by the Internal Revenue Service as a tax-exempt, charitable organization. Private funding indicates that the shelter receives private donations. Government contract indicates that the shelter has a contract with a municipality for animal control functions. Municipally funded indicates that the shelter receives funding from taxpayer dollars. ^c^ X indicates the shelter did not offer the program in 2020. ✓ indicates the shelter did offer the program in 2020. N/A represents missing data. ^d^ Animals from known COVID-19-positive homes were housed separately from the general shelter population for 14 days out of an abundance of caution per AVMA guidelines [8].

**Table 2:** Intake and outcome data for March–June 2019 and 2020.

	Dogs					Cats				
	*n Shelters*	2019	2020	% Change	*p*-Value	*n Shelters*	2019	2020	% Change	*p*-Value
**Intake**	12	248 (176)	137 (88)	−45	0.01 *	14	373 (293)	264 (209)	−29	0.02 *
Owner surrender	12	74 (60)	54 (41)	−27	0.03 *	14	154 (150)	107 (93)	−31	0.02 *
Stray/unowned	12	68 (92)	44 (56)	−35	0.01 *	14	163 (212)	130 (176)	−20	0.07
Transfer in	12	106 (128)	39 (61)	−63	0.03 *	14	56 (86)	27 (39)	−52	0.05
**Outcome**										
Adopted	12	181 (131)	97 (64)	−46	0.01 *	14	246 (194)	176 (125)	−28	0.03 *
Return to owner	12	35 (41)	24 (31)	−31	0.01 *	14	7 (10)	6 (7)	−14	0.54
Transfer out	12	6 (9)	3 (8)	−50	0.01 *	14	9 (27)	4 (11)	−56	0.23
Return to field	-	-	-	-	-	14	7 (13)	8 (15)	14	1.00
Euthanized	12	18 (37)	11 (25)	−39	0.04 *	14	28 (37)	17 (28)	−39	0.01 *
**Housing**										
Foster	8	52 (59)	38 (38)	−27	0.43	10	183 (220)	202 (230)	9	0.57
Exclusively in shelter ^a^	7	208 (108)	174 (103)	−16	0.34	9	371 (242)	272 (175)	−27	0.01 *

Data are shown as the mean *n* (standard deviation). * Indicates that there was a statistically significant difference (*p* < 0.05) between 2019 and 2020 based on paired sample *t*-tests (overall intake, adoption, housing) or Wilcoxon signed rank test (all other outcomes). ^a^ Housed exclusively in the shelter indicates that the animal did not spend any time in a foster care home.

**Table 3:** Shelter operations relative to COVID-19.

	Intake Types	Outcome Procedures	Foster Care Program	Community Programs
Shelter 1	X	✓	X	X
Shelter 2	X	X	✓	✓
Shelter 3	X	X	X	X
Shelter 4	✓	✓	X	✓
Shelter 5	✓	✓	✓	✓
Shelter 6	X	✓	X	✓
Shelter 7	✓	✓	✓	X
Shelter 8	✓	✓	✓	-
Shelter 9	✓	✓	X	✓
Shelter 10	X	✓	✓	✓
Shelter 11	X	X	X	-

X indicates that the shelter did not make a change to their operations between 2019 and 2020. ✓ indicates that the shelter made a change to their operations. Shelters 12, 13 and 14 did not provide data regarding their shelter operations.

## Data Availability

The data presented in this study are available in the Supplementary Material.

## Supplementary Materials

The following are available online at https://www.mdpi.com/article/10.3390/ani11092669/s1, Table S1: Canine intake and outcome data for March-June 2019 and 2020 (*n* = 12), Table S2: Feline intake and outcome data for March-June 2019 and 2020 (*n* = 14), Table S3: Changes to intake and outcome operations relative to COVID-19, Table S4: Changes to community programs relative to COVID-19.
