# Supplementary material for: Animal Shelters’ Response to the COVID-19 Pandemic: A Pilot Survey of 14 Shelters in the Northeastern United States
Source: Animals (Basel). 2021 Sep 10;11(9):2669. doi: 10.3390/ani11092669 (PMC8468430; doi:10.3390/ani11092669)
Supplement: Supplementary file 1 [file animals-11-02669-s001.zip › animals-1314341-supplementary.pdf]

Supplementary Table S1. Canine intake and outcome data for March-June 2019 and 2020 (*n* = 12)

		Intake			Outcome						Housing	
	Year	Owner surrender	Stray/ unowned	Transfer in	Adopted	Adoption %[a]	Return to owner	Transfer out	Euthanized	Euthanasia %[a]	Foster	Foster %[b]
**Shelter 1**	2019	36	0	269	302	99	2	8	5	2	69	23
	2020	39	0	188	206	91	1	0	7	3	105	46
	% change	8	0	-30	-32	-8	-50	-100	40	88	52	105
**Shelter 2**	2019	32	16	5	48	91	6	1	0	0	0	0
	2020	30	25	0	36	65	11	0	0	0	9	7
	% change	-6	56	-100	-25	-28	83	-100	0	0	N/A	N/A
**Shelter 3**	2019	62	0	0	71	115	0	0	0	0	-	-
	2020	58	0	0	60	103	0	0	0	0	-	-
	% change	-6	0	0	-15	-10	0	0	0	0	-	-
**Shelter 4**	2019	66	12	89	185	111	16	4	12	7	-	-
	2020	39	5	34	98	126	3	2	1	1	-	-
	% change	-41	-58	-62	-47	13	-81	-50	-92	-82	-	-
**Shelter 5**	2019	21	16	105	115	81	8	1	2	1	25	12
	2020	19	3	81	105	102	0	0	0	0	12	4
	% change	-10	-81	-23	-9	26	-100	-100	-100	-100	-52	-65
**Shelter 6**	2019	221	10	155	227	59	22	23	132	34	-	-
	2020	136	4	13	55	36	12	10	89	58	-	-
	% change	-38	-60	-92	-76	-39	-45	-57	-33	70	-	-
**Shelter 8**	2019	37	183	363	438	75	0	0	8	1	113	44
	2020	22	146	122	202	70	0	0	2	1	61	25
	% change	-41	-20	-66	-54	-7	0	0	-75	-50	-46	-44
**Shelter 10**	2019	93	305	1	203	51	128	26	29	7	163	31
	2020	113	159	0	164	60	93	27	12	4	48	13
	% change	22	-48	-100	-19	18	-27	4	-59	-39	-71	-59
**Shelter 11**	2019	143	100	1	106	43	85	3	7	3	40	100
	2020	91	80	0	62	36	72	0	4	2	63	100
	% change	-36	-20	-100	-42	-8	-15	-100	-43	-18	58	0
**Shelter 12**	2019	101	88	257	357	80	75	3	16	4	-	-
	2020	40	54	27	114	94	48	1	4	3	-	-

	% change	-60	-39	-89	-68	18	-36	-67	-75	-8	-	-
**Shelter 13**	2019	65	46	28	106	76	44	1	7	5	6	2
	2020	60	30	7	59	61	34	0	10	10	0	0
	% change	-8	-35	-75	-44	-20	-23	-100	43	105	-100	-100
**Shelter 14**	2019	7	38	1	14	30	31	1	0	0	0	0
	2020	0	20	0	8	40	18	0	0	0	4	13
	% change	-100	-47	-100	-43	31	-42	-100	0	0	N/A	N/A

Data show n cases between March-June unless indicated otherwise. [a] adoption and euthanasia rates calculated relative to intake, meaning rates may exceed 100% if more animals were adopted/euthanaized than entered the shelter during this period due to the existing shelter population. [b] foster care rates calculated relative to shelter population.

Supplementary Table S2. Feline intake and outcome data for March-June 2019 and 2020 (*n* = 14)

		Intake			Outcome							Housing	
		**Owner surrender**	**Stray/ unowned**	**Transfer in**	**Adopted**	**Adopted %[a]**	**Return to owner**	**Transfer out**	**Return to field**	**Euthanized**	**Euthanasia % [a]**	**Foster**	**Foster % [b]**
**Shelter 1**	*2019*	231	0	178	306	75	6	0	0	11	3	267	65
	2020	197	0	106	209	69	10	4	0	6	2	275	77
	% change	-15	0	-40	-32	-8	67	N/A	0	-45	-26	3	19
**Shelter 2**	*2019*	2	51	0	48	91	0	0	0	0	0	0	0
	2020	0	70	1	58	82	0	0	0	4	6	20	13
	% change	-100	37	N/A	21	-10	0	0	0	n/a	n/a	n/a	n/a
**Shelter 3**	*2019*	269	0	0	194	72	0	0	0	0	0	-	-
	2020	250	0	0	176	70	0	0	0	0	0	-	-
	% change	-7	0	0	-9	-2	0	0	0	0	0	-	-
**Shelter 4**	*2019*	411	39	90	411	76	10	4	2	13	2	-	-
	2020	151	33	38	289	130	0	1	1	9	4	-	-
	% change	-63	-15	-58	-30	71	-100	-75	-50	-31	68	-	-
**Shelter 5**	*2019*	12	74	0	89	103	2	1	27	11	13	70	9
	2020	9	56	17	61	74	0	0	0	4	5	99	16
	% change	-25	-24	N/A	-31	-28	-100	-100	-100	-64	-62	41	75
**Shelter 6**	*2019*	445	191	226	672	78	28	6	0	123	14	-	-
	2020	230	58	94	297	78	19	5	0	104	27	-	-
	% change	-48	-70	-58	-56	0	-32	-17	0	-15	91	-	-
**Shelter 7**	*2019*	1	47	0	33	69	0	0	1	3	6	-	-
	2020	4	52	0	27	48	0	0	0	9	16	-	-
	% change	300	11	0	-18	-30	0	0	-100	200	157	-	-
**Shelter 8**	*2019*	50	593	49	411	59	0	0	7	37	5	223	27
	2020	74	587	30	392	57	0	0	10	18	3	293	43
	% change	48	-1	-39	-5	-4	0	0	43	-51	-51	31	61
**Shelter 9**	*2019*	45	39	24	98	91	0	4	0	0	0	64	31
	2020	22	37	8	62	93	2	1	0	0	0	30	25
	% change	-51	-5	-67	-37	2	N/A	-75	0	0	0	-53	-20
**Shelter 10**	*2019*	226	671	3	444	49	10	102	40	77	9	683	56

	2020	223	439	0	384	58	15	40	18	49	7	522	56
	% change	-1	-35	-100	-14	18	50	-61	-55	-36	-14	-24	1
**Shelter 11**	*2019*	119	276	0	141	36	8	1	1	70	18	395	100
	2020	117	241	0	126	35	7	0	46	8	2	649	100
	% change	-2	-13	0	-11	-1	-13	-100	4500	-89	-87	64	0
**Shelter 12**	*2019*	129	110	216	379	83	11	1	0	13	3	-	-
	2020	75	49	84	160	77	14	0	0	6	3	-	-
	% change	-42	-55	-61	-58	-8	27	-100	0	-54	1	-	-
**Shelter 13**	*2019*	210	121	0	186	56	28	0	22	22	7	126	17
	2020	134	150	3	189	66	11	1	34	22	8	132	23
	% change	-36	24	N/A	2	17	-61	N/A	55	0	15	5	30
**Shelter 14**	*2019*	6	74	0	32	40	1	0	0	11	14	-	-
	2020	5	54	0	38	64	0	1	0	4	7	-	-
	% change	-17	-27	0	19	61	-100	N/A	0	-64	-51	-	-

Data show n cases between March-June unless indicated otherwise. [a] adoption and euthanasia rates calculated relative to intake, meaning rates may exceed 100% if more animals were adopted/euthanaized than entered the shelter during this period due to the existing shelter population. [b] foster care rates calculated relative to shelter population.

Supplementary Table S3. Changes to intake and outcome operations relative to COVID-19.

	Shelters																					
	1		2		3		4		5		6		7		8		9		10		11	
	'19	'20	'19	'20	'19	'20	'19	'20	'19	'20	'19	'20	'19	'20	'19	'20	'19	'20	'19	'20	'19	'20
Intake																						
Intake by appointment	✓	✓	✓	✓	✓	✓	✓	✓	✓	✓	✓	✓	X	✓	-	-	✓	✓	✓	✓	-	-
Owner surrenders	✓	✓	✓	✓	-	-	✓	✓	✓	✓	✓	✓	-	-	✓	✓	✓	✓	✓	✓	X	X
Sick/injured dogs and cats	X	X	✓	✓	-	-	✓	✓	✓	✓	✓	✓	-	-	✓	✓	X	✓	✓	✓	✓	✓
Healthy stray dogs	X	X	✓	✓	-	-	✓	✓	✓	✓	X	X	-	-	✓	✓	X	X	✓	✓	X	X
Healthy stray puppies	X	X	✓	✓	-	-	✓	✓	✓	✓	X	X	-	-	✓	✓	X	X	✓	✓	X	X
Healthy stray cats	X	X	✓	✓	-	-	✓	✓	✓	✓	✓	✓	-	-	✓	✓	✓	✓	✓	✓	X	X
Healthy stray kittens	✓	✓	✓	✓	-	-	✓	✓	✓	✓	✓	✓	-	-	✓	✓	✓	✓	✓	✓	X	X
Pick-up/trap pets of owners in crisis	X	X	X	X	-	-	✓	X	X	✓	X	X	-	-	X	✓	X	X	✓	✓	X	X
Pick-up/trap non-aggressive animals	X	X	X	X	-	-	X	X	X	X	X	X	-	-	X	✓	X	X	✓	✓	X	X
Pick-up/trap aggressive animals	X	X	X	X	-	-	X	X	X	X	X	X	-	-	✓	✓	X	X	✓	✓	X	X
Outcomes																						
**Adoption**																						
Adoption by appointment	X	✓	✓	✓	✓	✓	X	✓	X	✓	X	✓	X	✓	-	-	X	✓	X	✓	-	-
Adoption ambassadors	X	✓	✓	✓	✓	✓	X	X	X	X	✓	✓	X	✓	-	-	✓	✓	✓	✓	-	-
Open adoptions program	✓	✓	✓	✓	X	X	✓	✓	X	X	✓	✓	X	X	-	-	✓	✓	✓	✓	-	-
Open selection program	X	X	X	X	X	X	X	X	X	X	X	X	X	X	-	-	X	X	X	X	-	-
Spay/neuter prior to adoption	✓	✓	✓	✓	-	-	✓	X	✓	X	✓	X	-	-	✓	✓	✓	✓	✓	✓	X	X
Animal adopted before spay/neuter	X	X	X	X	-	-	X	✓	X	✓	X	✓	-	-	✓	X	X	X	X	X	X	X
**Return to owner/field**																						
Return to owner	X	X	✓	✓	-	-	✓	✓	✓	✓	✓	✓	-	-	✓	✓	X	X	✓	✓	X	X
Return-to-field for adult cats	X	X	X	X	-	-	✓	X	✓	X	✓	✓	-	-	X	✓	✓	✓	✓	✓	X	X
Foster care																						
Finder-to-foster program	X	X	X	X	X	X	X	X	✓	✓	X	X	X	X	-	-	✓	✓	X	✓	-	-
Foster-on-deck (waitlist of carers)	✓	✓	X	X	X	X	X	X	X	✓	X	X	X	✓	-	-	X	X	X	X	-	-
Spay/neuter prior to foster	X	X	✓	✓	-	-	X	X	✓	X	✓	✓	-	-	✓	✓	X	X	✓	✓	X	X
Animal sent to foster before spay/neuter	✓	✓	X	✓	-	-	✓	✓	X	X	✓	✓	-	-	✓	X	✓	✓	✓	✓	X	X

X indicates the shelter did not offer the program in 2019 or 2020. ✓ indicates the shelter did offer the program in 2019 or 2020. Red text shows a change between 2019 and 2020. Shelters 12, 13 and 14 did not provide data regarding their shelter operations.

Supplementary Table S4. Changes to community programs relative to COVID-19.

	**Shelters**																					
	**1**		**2**		**3**		**4**		**5**		**6**		**7**		**8**		**9**		**10**		**11**	
	**'19**	**'20**	**'19**	**'20**	**'19**	**'20**	**'19**	**'20**	**'19**	**'20**	**'19**	**'20**	**'19**	**'20**	**'19**	**'20**	**'19**	**'20**	**'19**	**'20**	**'19**	**'20**
**Community programs**																						
Subsidized veterinary care	✓	✓	✓	✓	X	X	✓	X	X	X	✓	X	X	X	-	-	✓	✓	X	X	-	-
Pet food pantry	✓	✓	✓	X	✓	✓	✓	✓	✓	✓	✓	✓	X	X	-	-	X	✓	✓	✓	-	-
Pet retention program	✓	✓	✓	✓	X	X	✓	✓	✓	✓	X	X	X	X	-	-	✓	✓	✓	✓	-	-
Education program to help owners troubleshoot problems to retain pet	✓	✓	✓	✓	X	X	✓	✓	✓	✓	X	X	X	X	-	-	✓	✓	X	✓	-	-
**Other**																						
Public campaign to discourage intake of certain populations	X	X	✓	✓	X	X	X	X	X	✓	X	X	X	X	-	-	X	✓	X	✓	-	-
Written disaster or emergency response plan	X	X	✓	✓	X	X	X	X	X	✓	X	X	X	X	-	-	X	X	X	✓	-	-

X indicates the shelter did not offer the program in 2019 or 2020. ✓ indicates the shelter did offer the program in 2019 or 2020. Red text shows a change between 2019 and 2020. Shelters 12, 13 and 14 did not provide data regarding their shelter operations.
